# Utilization of Phytic Acid by Cooperative Interaction in Rhizosphere

**DOI:** 10.1264/jsme2.ME2801rh

**Published:** 2013-03-12

**Authors:** Masahito Hayatsu

**Affiliations:** 1 National Institute for Agro-Environmental Sciences, 3-1-3 Kannondai, Tsukuba 305-8604, Japan

Plants and microbes interact in various relationships which have both adverse and beneficial effects on plant growth and microbial communities. Many processes of these interactions occur at or near the root–soil interface, which is known as the rhizosphere. The rhizosphere is defined as the soil region under the immediate influence of plant roots. Many studies suggest that the microbial community in rhizosphere inhibits invasion of the plant pathogen to the plant root (10, 14, 22) and play an important role in essential reactions of the nutrient cycle (6, 7, 9). Microorganisms degrade organic material and release inorganic nutrients such as ammonium and phosphate, which can then be taken up by plants. Microorganisms involved in phosphorus metabolism in the rhizosphere have received considerable attention because plant-available phosphorus is limited in many soils. In the current issue, Unno and Shinano report phytic acid degradation by a rhizosphere specific microbial community (18).

Most agricultural soils contain too little available phosphorus for high and stable crop production (5). Therefore large amounts of phosphorus fertilizers are applied to agricultural fields to maintain high levels of crop productivity. Phosphorus fertilizers are usually made from phosphate rock which has been suggested to be depleted in the 21st century (2).

A large portion of phosphorus in most soils is organic forms, and a major component of soil organic phosphorus is phytic acid mainly consisting of inositol penta- and hexaphosphates (3). Most plant species cannot utilize soil phytic acid as a phosphorus source (Fig. 1), because they lack extracellular phytases which are enzymes that can liberate inorganic phosphate from the phytic acid. Phytases are produced by many microorganisms including bacteria, yeasts and fungi (4). Microbial degradation of phytic acid is presumed to occur in soils because phytase-producing microorganisms are widely distributed in various soils (12). In several laboratory studies, inoculation of phytase-producing bacteria to plants increased the phosphorus availability for plants supplied with phytic acid (12).

In a previous work, Unno *et al.* focused on phytic acid metabolism in the rhizosphere of *Lupinus albus* and isolated about 300 strains of phytic acid-utilizing bacteria from the rhizosphere (17). In this experiment, they used a unique field soil, which had not received phosphate fertilizer for 90 years. Almost all isolated bacteria were identified as *Burkholderia* genus on the basis of 16S rDNA sequence analysis. This is the first evidence that *Burkholderia* has phytase activity and shows plant growth promoting effect with phytic acid as the sole phosphorus source. *Burkholderia* is a genus commonly found in soil and associated with plants. Many *Burkholderia* strains have been shown to provide benefits to plant, through nitrogen supply by nitrogen fixation and suppression of plant disease (15).

Unno and Shinano conducted culture experiments of *Lotus japonicus* using the unique field soil which was used for isolation of phytase producing *Burkholderia* strains (17), to investigate adaptation of the microbial community to phytic acid degradation in the rhizosphere. They found that the rhizosphere of *L. japonicus* showed significant phosphorus utilization from phytic acid applied to the soil under certain conditions. They used molecular fingerprint analysis (DGGE) and metagenomic analysis using pyrosequencing to identify key microbes and to elucidate the processes involved in phytic acid degradation in the rhizosphere. The DGGE analysis showed no clear relationship between the microbial composition and the phytic acid utilization of *L. japonicas.* Although DGGE analysis is a powerful tool to evaluate the microbial composition in soil environments (1, 13, 16, 19), it has some major limitations such as low sensitivity in detecting rare members of the microbial community and co-migration of distinct gene sequences in single bands containing multiple sequences. However, recently metagenomic analysis using pyrosequencing is improving the understanding of gene diversity in microbial communities in various environments (8, 11). There are a few studies using metagenomic analysis for functional microbial community in rhizosphere. Unno and Shinano have successfully used metagenomic analysis to elucidate the diversity of the microbial community and to identify the genes encoding enzymes potentially involved in phytic acid degradation in the rhizosphere. Comparison of metagenomic data from phytic acid utilizing-rhizosphere microbial community and non-phytic acid utilizing-rhizosphere microbial community revealed changes in the relative abundance of some bacterial classes that included strains that potentially promote plant growth and phytic acid utilization. Metagenomic analysis also showed changes in the relative abundance of some genes encoding enzymes associated with phytic acid utilization. These phylogenetic and metabolic properties suggest that bacterial communities adapted to degradation and utilization of phytic acid in the rhizosphere under the influence of plant root. These results provide new insights beyond individual bacterial biochemical and genetic information on phytic acid metabolism.

Although metagenomic DNA-based analysis provides useful information on the phylogenetic and metabolic genes of a microbial community, it cannot be used to estimate the active microbial population size and the expression level of target genes. Metatranscriptomic analysis has significant potential for describing functional processes in complex microbial communities. Analysis of mRNA and 16S rRNA can reveal metabolically active populations. In the past several years, some attempts have been made to investigate microbial gene expression in soils (20), and recent advances in the procedure of extraction and purification of soil mRNA (21) make it possible to elucidate the functional status of microbial community in soil ecosystems including rhizospheres. Such metatranscriptomic approaches could provide significant insights into the mechanisms of phytic acid degradation and utilization of phosphate in the rhizosphere. Further understanding of the roles of microbial consortia in rhizosphere and plant roots in the establishment of the phytic acid utilization system could be exploited to improve agricultural management practices in crop nutrition.

## Figures and Tables

**Fig. 1:**
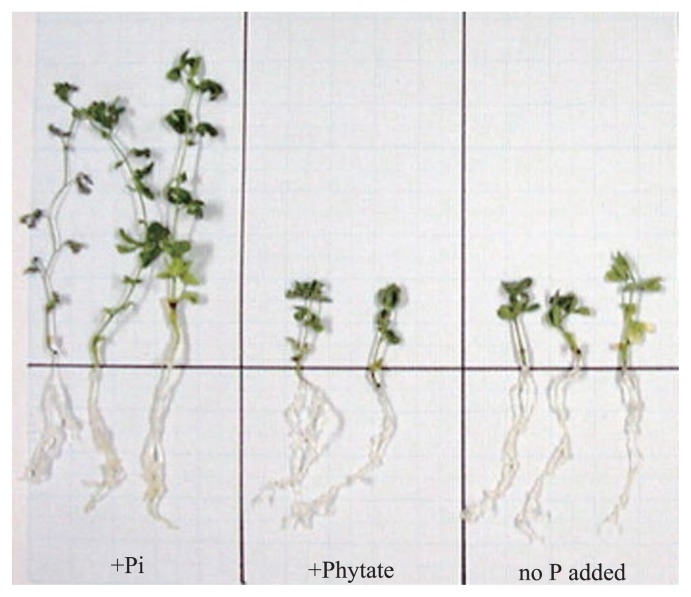
Seedlings of *Lotus japonicus* grown in the presence of inorganic phosphate, phytic acid and in the absence of phosphate (Y. Unno and T. Shinano, unpublished data).
